# COVID-19 infection epidemic: the medical management strategies in Heilongjiang Province, China

**DOI:** 10.1186/s13054-020-2832-8

**Published:** 2020-03-18

**Authors:** Hongliang Wang, Sicong Wang, Kaijiang Yu

**Affiliations:** 1 Department of Critical Care Medicine, The Second Affiliated Hospital of Harbin Medical University, Harbin, Heilongjiang China; 2 Heilongjiang Province Intensive Care Medical Aid Group for CVOID-19, Wuhan, Hubei China; 3 Department of Critical Care Medicine, The Cancer Hospital of Harbin Medical University, Harbin, Heilongjiang China; 4 Department of Critical Care Medicine, The First Affiliated Hospital of Harbin Medical University, No. 23, Youzheng Road, Nangang District, Harbin, Heilongjiang China; 5 Expert Panel of Heilongjiang COVID-19, Harbin, Heilongjiang China

In late December 2019, an outbreak of the 2019-novel coronavirus (COVID-19) caused a substantial public health crisis in Wuhan, China, and then expeditiously spread all over China [1–3]. As of March 4, 2020, 80,409 cases of COVID-19 had been confirmed in mainland China [4]. While in Heilongjiang province, which locates in northeastern China with 38.24 million residents and an area of 473,000 km^2^, all of its 13 cities were affected, making it one of the most serious areas for the outbreak of COVID-19 in China. Up to February 23, 2020, there were 480 confirmed cases of COVID-19; however, no newly diagnosed cases since then. Most of the infected patients were male and there were 13 deaths (2.7%). A series of protocols had been established since the first confirmed case emerged, and we herein summarize our experience from Heilongjiang province in dealing with COVID-19.

## Protection of medical staffs

### Education and training of staffs

As soon as the outbreak of COVID-19 began in Wuhan, the Heilongjiang provincial health administration department started to launch training protocols for all the medical staffs. The content of training included personal protection such as hand hygiene, wearing personal protective equipment (PPE), safe waste disposal, and emergency handling protocols. The means of training included presentations, videos, and WeChat. First, the infection control experts from various hospitals trained their own staffs both theoretically and practically. The training emphasized the importance of standardized protection. Second, the infection control experts conducted standardized pre-job training. The infection control experts supervised the whole process to ensure that all the staffs had followed the correct procedures. Third, the infection control experts monitored the entire process before and after the medical staff entering the isolation wards. They also supervised the procedures of medical staffs in the isolation wards by wireless communication equipment.

### Mental health care

The mental stress of the medical staffs increased significantly since they had to work in a relatively confined space, wearing thick isolation clothes, and care for a large number of anxious patients. The high-intensity work further deteriorated the mental health of the medical staffs, especially for those who came from all over the country to support Wuhan. It is crucial to ensure the stable psychological state of the medical staffs. Therefore, early professional intervention is necessary. At the same time, active communication with family members and initiative support from a local hospital can also be very helpful.

### Monitoring of physical condition

It is particularly important to ensure the physical health of the medical staffs. All of them should undergo medical examinations, including blood routine tests and chest CT before managing COVID-19 patients. Daily self-examinations of respiratory symptoms and body temperature were also conducted. Once the medical staff had any uncomfortable symptoms, they should report to the team leader immediately. A specialized screening for COVID-19 would be performed. The medical staffs who treated COVID-19 patients lived in special and isolated areas.

### Management of protective equipment

Adequate protective equipment was prepared for all the medical staffs who participated in the management of COVID-19. The protective equipment included medical masks, goggles, face shields, and waterproof isolation clothes. The protective equipment was supplied by the government and hospitals, as well as donated by the public. The equipment such as goggles and face shields that was reused would be disinfected strictly.

## Reassignment of medical resources

**Fig. 1:**
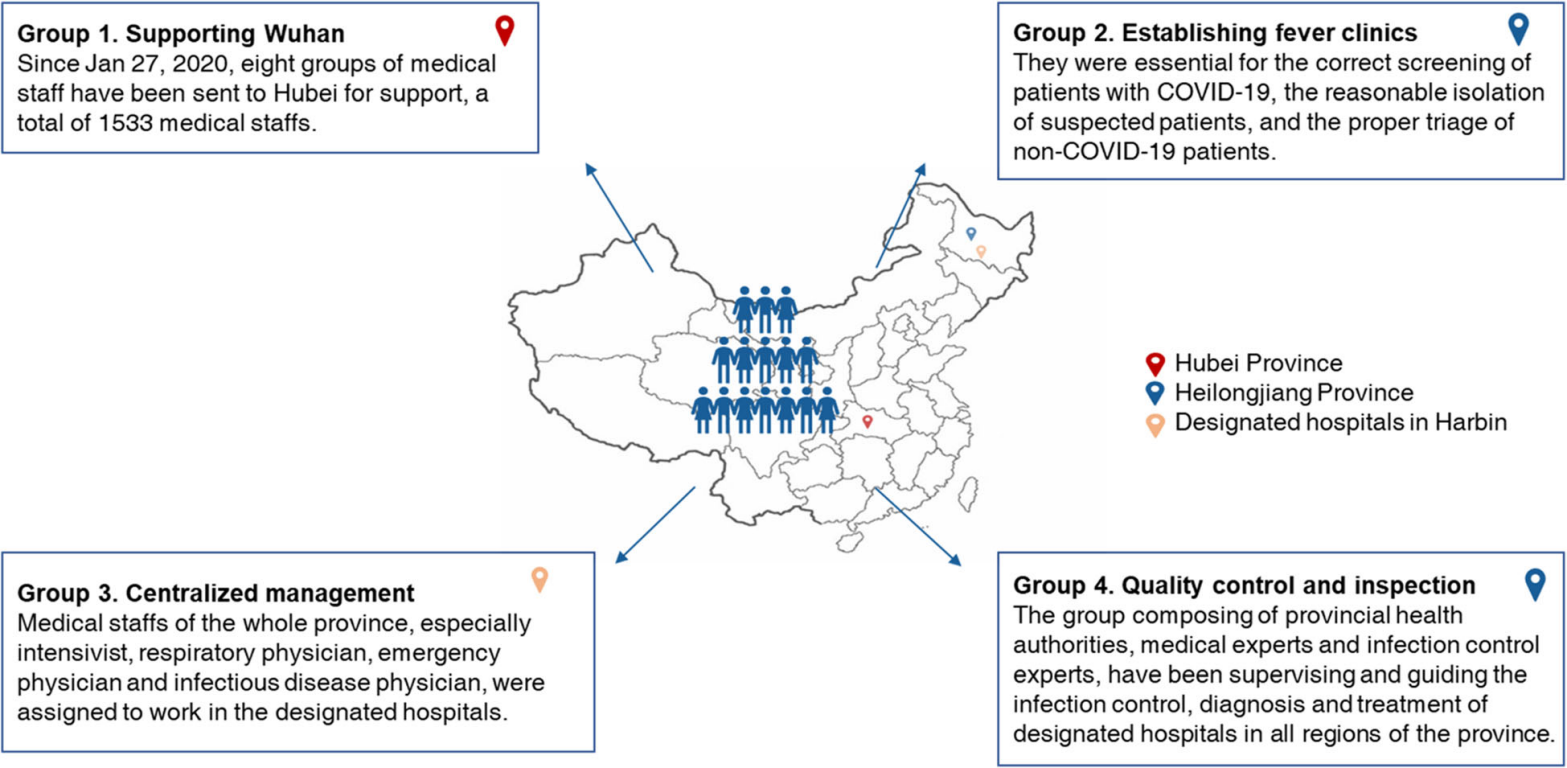
Reassignment of medical resources in Heilongjiang province. *COVID-19: Coronavirus Disease 2019

Heilongjiang province set up a multidisciplinary team (MDT) soon after the outbreak of COVID-19, including intensive care unit (ICU), emergency department, infectious disease department, respiratory department, psychological department, infection control department, administrative department, and nursing department. Meanwhile, the medical resources of the whole province were redistributed to meet medical needs. Medical staffs in Heilongjiang province were divided into 4 groups (Fig. 1).

### Group 1: Supporting Wuhan

Wuhan, as the most serious epidemic area, needs a national support. The first set of medical teams arrived in Wuhan on January 27, 2020, and soon started medical work. As of February 23, Heilongjiang province had dispatched eight medical teams consisting of 1533 medical staffs to Hubei Province.

### Group 2: Establishing fever clinics

The majority of the medical staffs worked in the fever clinics, which involved the largest number of patients to be screened. Fever clinics were essential for the proper triage of fever patients: diagnosis of COVID-19 patients, isolation of suspected cases, and exclusion of non-COVID-19 patients.

### Group 3: Centralized management

Heilongjiang province appointed four designated hospitals in Harbin to treat all the COVID-19 patients. Medical staffs of the whole province, especially intensivist, respiratory physician, emergency physician, and infectious disease physician, were assigned to work in the designated hospitals.

### Group 4: Quality control and inspection group

The group members included provincial health authorities, medical experts, and infection control experts. During inspection, the team summarized, discussed, and handled any problems timely, thus ensuring that the infection rate of the medical staffs was zero.

## Plans for hierarchical treatment

### Procedures of hierarchical treatment

**Fig. 2:**
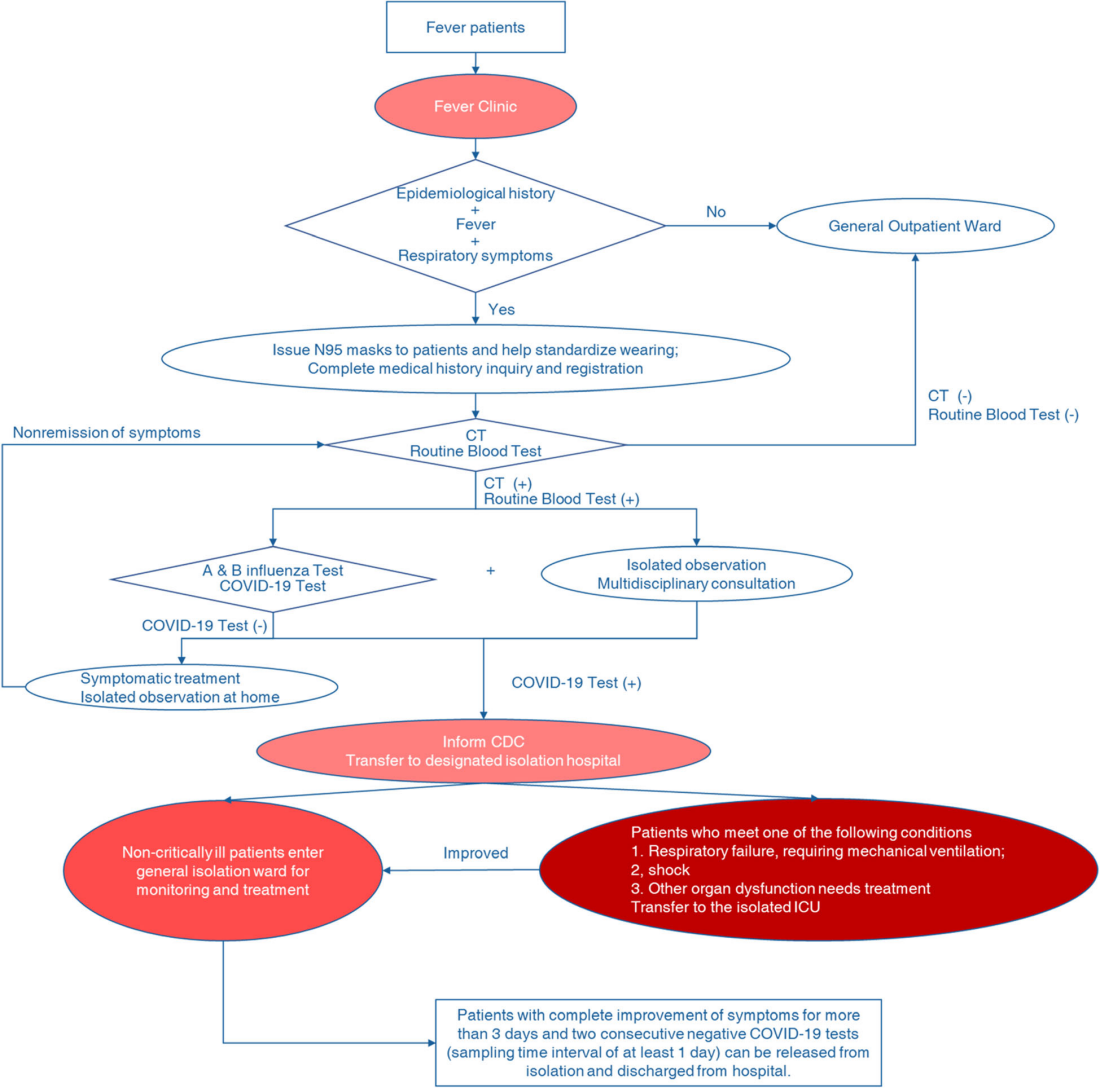
Treatment procedures of patients with suspected COVID-19. *COVID-19, Coronavirus Disease 2019; ICU, intensive care unit; CDC, Centers for Disease Control and Prevention

During the outbreak of COVID-19, all the patients with fever in our province were screened according to the procedure, as detailed in Fig. 2.

### Strategies for management and reassignment of medical staffs

In all isolation wards, a three-level round system was adopted, both in Heilongjiang and Hubei. The morning and evening medical care shifts were adopted to achieve the standardized management of COVID-19. The medical resources were reassigned accordingly: physicians from general wards who specialized in respiratory or infectious disease treated mild patients, while critical cases were handled by a team led by intensivists. Medical treatment and nursing were managed in the same way as mentioned above.

## Utilization of network platform

To prevent the spread of COVID-19, our province suspended outpatient services and non-emergency surgeries of all levels of hospitals. Health Commission of Heilongjiang Province launched online free consultations. More than 12,000 medical staffs provided free online consultation, initial screening, popularizing the knowledge, and summarizing the experiences of managing COVID-19.

In conclusion, the COVID-19 outbreak is a significant threat to international health and a big challenge for all of us. We need to understand more and more about the disease to overcome it.

## Data Availability

All data generated or analyzed during this study are included in this published article.
